# The Physiological Functions of Universal Stress Proteins and Their Molecular Mechanism to Protect Plants From Environmental Stresses

**DOI:** 10.3389/fpls.2019.00750

**Published:** 2019-06-05

**Authors:** Yong Hun Chi, Sung Sun Koo, Hun Taek Oh, Eun Seon Lee, Joung Hun Park, Kieu Anh Thi Phan, Seong Dong Wi, Su Bin Bae, Seol Ki Paeng, Ho Byoung Chae, Chang Ho Kang, Min Gab Kim, Woe-Yeon Kim, Dae-Jin Yun, Sang Yeol Lee

**Affiliations:** ^1^Division of Applied Life Science (BK21Plus), Plant Molecular Biology and Biotechnology Research Center, Gyeongsang National University, Jinju, South Korea; ^2^College of Pharmacy and Research Institute of Pharmaceutical Science, Gyeongsang National University, Jinju, South Korea; ^3^Institute of Agricultural and Life Science (IALS), Gyeongsang National University, Jinju, South Korea; ^4^Department of Biomedical Science and Engineering, Konkuk University, Seoul, South Korea

**Keywords:** abiotic/biotic defense signaling, biotechnological application, external stress, molecular mechanism of USPs, multi-functional roles, universal stress protein

## Abstract

Since the original discovery of a Universal Stress Protein (USP) in *Escherichia coli*, a number of USPs have been identified from diverse sources including archaea, bacteria, plants, and metazoans. As their name implies, these proteins participate in a broad range of cellular responses to biotic and abiotic stresses. Their physiological functions are associated with ion scavenging, hypoxia responses, cellular mobility, and regulation of cell growth and development. Consistent with their roles in resistance to multiple stresses, USPs show a wide range of structural diversity that results from the diverse range of other functional motifs fused with the USP domain. As well as providing structural diversity, these catalytic motifs are responsible for the diverse biochemical properties of USPs and enable them to act in a number of cellular signaling transducers and metabolic regulators. Despite the importance of USP function in many organisms, the molecular mechanisms by which USPs protect cells and provide stress resistance remain largely unknown. This review addresses the diverse roles of USPs in plants and how the proteins enable plants to resist against multiple stresses in ever-changing environment. Bioinformatic tools used for the collection of a set of USPs from various plant species provide more than 2,100 USPs and their functional diversity in plant physiology. Data from previous studies are used to understand how the biochemical activity of plant USPs modulates biotic and abiotic stress signaling. As USPs interact with the redox protein, thioredoxin, in Arabidopsis and reactive oxygen species (ROS) regulates the activity of USPs, the involvement of USPs in redox-mediated defense signaling is also considered. Finally, this review discusses the biotechnological application of USPs in an agricultural context by considering the development of novel stress-resistant crops through manipulating the expression of *USP* genes.

## Introduction

**FIGURE 1:**
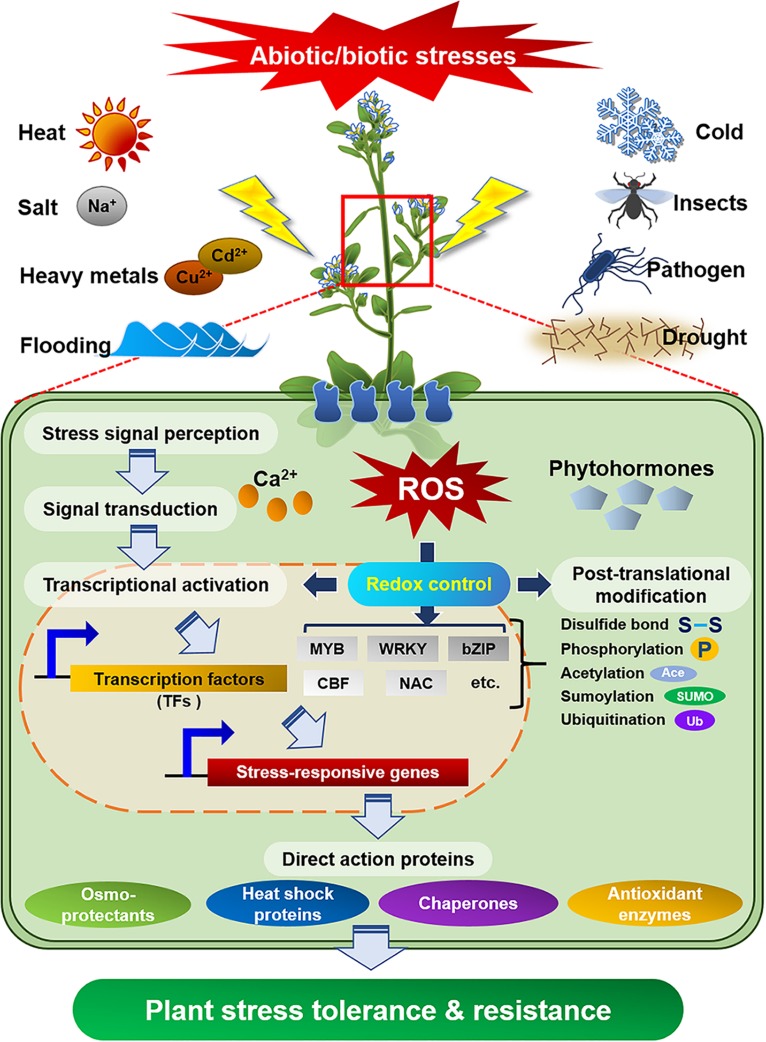
Defense signaling in plants against diverse abiotic/biotic external stresses. Specific receptors in plant cells perceive external stresses and transduce the signal into downstream components, which activate or express defense molecules to protect plants from the stresses.

Plants as sessile organisms are persistently confronted with detrimental factors that are arisen from ever-changing environment. To cope with environmental stresses that are harmful to their growth and development, plants have evolved sophisticated and delicate defense mechanisms. In fact, the external stress activates diverse defense signaling that include the production of reactive oxygen species (ROS), change in redox potential or cellular level of Ca^2+^ ion, disruption of ion homeostasis, and adjustment of membrane fluidity (Gilroy et al., 2016; Choudhury et al., 2017). After sensing the external stress *via* specific receptors, plants transduce the foreign signal into intracellular downstream signaling pathways including the activation of protein kinase or phosphatase, stimulation of downstream target proteins, and biosynthesis of phytohormones for the control of plant growth/development (Figure 1; Sheikh et al., 2016; Akimoto-Tomiyama et al., 2018). In particular, cross-talk of these complex signaling networks precisely regulates the expression of stress responsive genes and protects plants from external stresses (Sewelam et al., 2016; Choudhury et al., 2017; Nejat and Mantri, 2017). Thus, the identification of diverse stress-resistant genes/proteins from various organisms and elucidation of their biochemical and physiological functions can provide valuable information for the preparation of valuable crops with stress tolerance and high productivity.

As a representative defense protein that protects host organisms from diverse external stresses, a novel stress-inducible protein with an estimated molecular weight of 13.5 kDa was identified from the cytosolic fraction of *E. coli* using matrix-assisted laser desorption/ionization (MALDI) analysis (VanBogelen et al., 1990). The protein designated ‘Universal Stress Protein (USP)’ is significantly overexpressed under unfavorable environmental stresses, such as nutrients starvation (deficiency of carbon, nitrogen, phosphate, sulfate, and amino acids), heat/cold shock, oxidative stress, heavy metal toxicity, uncoupler of electron transport chains, exposure to polymyxin, cycloserine, ethanol and antibiotics etc. (VanBogelen et al., 1990; Kvint et al., 2003). Following the discovery of USP from *E. coli*, many USP proteins containing at least one USP domain consisting of 140 to 160 conserved amino acid residues with other diverse functional motifs have been found from a wide variety of organisms including bacteria, archaea, plants, and metazoans (Foret et al., 2011; Vollmer and Bark, 2018). The USP domain (Pfam accession number PF00582) forming an α/β subdomain structure is important for numbers of cellular defense signaling (Kerk et al., 2003; Kvint et al., 2003; Tkaczuk et al., 2013) and numerous stress-resistant metabolic pathways (VanBogelen et al., 1990; Nystrom and Neidhardt, 1992; Zarembinski et al., 1998; Ndimba et al., 2005; Siegele, 2005; Persson et al., 2007; Sen et al., 2019). The functions of USPs are shown to involve in protein scaffolding, holding and preventing the denaturation of molten globular macromolecules, and cellular protein transport (Vollmer and Bark, 2018). Moreover, several USPs exhibit DNA binding, repairing, and refolding activities that can support organisms to protect their nucleic acids from external stresses (Kvint et al., 2003; Drumm et al., 2009).

Consistent with their multi-functional roles, USPs possess a variety of other functional motifs and thus show a high degree of structural diversities. USP-like protein groups also include flavoproteins, which are involved in electron transport, N-type protein phosphatase, and ATP sulfhydrylases (Aravind et al., 2002). Based on their structural homology with X-ray crystal structure of MJ0577 protein isolated from *Methanocaldococcus jannaschii* or with the protein structure of USPA from *Haemophilus influenza*, USPs are largely classified into two categories (Sousa and McKay, 2001). USPs belonging to the first group contain an ATP-binding motif at their C-terminal region [G-2X-G-9X-(S/T)] and have an α/β-core structure consisting of five β-strands and four α-helical structures (Aravind et al., 2002). By contrast, polypeptides in the second group are lacking the ATP-binding residues and unable to bind or utilize ATP (Sousa and McKay, 2001). The structural and functional diversity of USPs results in many orthologous proteins being placed in USP groups, producing a large USP superfamily (Siegele, 2005).

Whereas the physiological function and structural diversity of USPs have been extensively investigated in microorganisms, only a few studies have been made in plants, although plants also contain large numbers of USPs. Therefore, in this paper, we will examine the biochemical and molecular properties, structural characteristics, and functional diversities of plant USPs, after reviewing the bacterial USP properties. Considering the redox-mediated control of its chaperone activity (Jung et al., 2015), particular attention will also be paid to the redox-dependent functional and conformational regulation. To the best of our knowledge, this is the first review paper of plant USPs that will serve much valuable information to the plant biologists for analyzing their molecular mechanisms and development of stress tolerant crops with high productivity. Therefore, in the final section, we are focusing on their biotechnological application in agricultural research fields.

### Functional and Structural Diversity of Bacterial USPs

Following the determination of amino acid sequence and protein structure of the first USP in *E. coli*, large numbers of USP homologs have been identified from bacterial sources and formed large USP families (Kvint et al., 2003). The *E. coli* USPs contain six different proteins including USPA, USPC, USPD, USPE, USPF and USPG. In fact, USPB was identified from stress condition and named USPB, of which gene was located immediately upstream of *USPA* gene (Farewell et al., 1998). However, USPB was not considered as a bona fide *E. coli* USP and eliminated from USP groups, because the protein was shown to be an integral membrane protein with two putative transmembrane domains and the molecular structure of the protein did not satisfy the criteria of USP structures. Finally, USPB is missed from the *E. coli* USP groups (Farewell et al., 1998; Tkaczuk et al., 2013; Vollmer and Bark, 2018). The six *E. coli* USPs are classified into four subclasses based on their structural similarity and amino acid sequence homology as follows. Whereas Class I without having the ATP binding motif includes USPA, USPC and USPD, Class II containing ATP binding motif is composed of USPF and USPG. In contrast to Class I & II, USPE in *E. coli* has tandem-repeated two USP domains in a polypeptide, that are designated E1 and E2 domains corresponding to the first and second USP domains, respectively. The E1 and E2 domains of *E. coli* USPE are grouped into Class III and Class IV.

**FIGURE 2:**
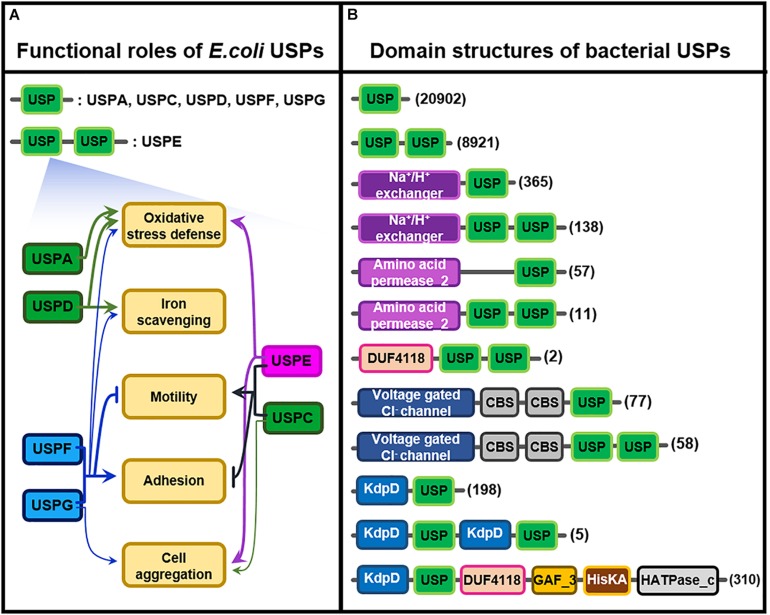
Functional roles of *E. coli* USPs and molecular structures of diverse bacterial USPs. **(A)** Functional roles of the six different *E. coli* USPs containing USPA, USPC, USPD, USPE, USPF, and USPG. Thick and thin arrows indicate the major and minor roles of specific USPs, respectively. T-shape arrows represent suppression of the physiological responses. USPs linked by brackets share the common physiological roles. This Figure 2A is modified from the reference of Nachin et al. (2005). **(B)** Molecular structures of the diverse bacterial USPs containing only a USP domain or USP domains fused with other catalytic motifs that are obtained from Pfam database (https://pfam.xfam.org/family/Usp). The numbers of USPs having the specific type of molecular structure are indicated at the right side of the figure in parenthesis. Each type of domain and motif is represented by different color-boxes.

*E. coli* USP belonging to each subclass takes its own specific function in particular environmental stress, as shown in Figure 2A (Kvint et al., 2003; Nachin et al., 2005); USPA and USPD in Class I play their roles in the resistance against oxidative stress and iron scavenging, but USPF and USPG protein in Class II also partly participate in the protection of bacterial cells from the same oxidative stress. Thus *USPD* mutant exhibits a high sensitivity to streptonigrin, causing an increase in intracellular iron concentration, which suggests that USPD plays a role in cellular iron scavenging. In addition to their anti-oxidative function, USPC and USPE, F, and G play in cellular adhesion, agglutination, cell motility, and swimming (Nachin et al., 2005). In contrast to the roles played by USPC and USPE, USPF and USPG belonging to Class II played different functions in cellular migration or movement. They negatively regulate bacterial mobility but positively control cell affixment and agglomeration. These results clearly indicate that the functions of various bacterial USPs are coordinated to enhance the stress tolerance of cells against harsh external circumstances.

The multiple functions of USPs in other bacteria are derived from their structural diversity. During the process of evolution, the USP domain is probably fused with other catalytic motifs to produce multi-structural USP proteins having diverse biochemical and molecular functions. Therefore, in addition to their single USP domain, USPs contain highly divergent other functional motifs, including protein kinase, Na^+^/H^+^ exchanger, and amino acid permease motifs, as well as a voltage gated Cl^–^ channel, whose function have not yet been clarified (Figure 2B; Kvint et al., 2003; Tkaczuk et al., 2013). The specific property of individual USPs under stress conditions might be dependent on their fused catalytic motifs. Thus, the combination of USP domain with other specific catalytic motif is likely to produce various functions that can protect host organisms from diverse external stresses. In reality, functional diversity of bacterial USPs has been demonstrated as follows; USP in *Acinetobacter baumannii*, a bacterium causing pneumonia and sepsis, is essential for protecting the pathogen from oxidative stress and respiratory toxins (Elhosseiny et al., 2015; O’Connor and McClean, 2017). Similarly, USP from *Salmonella typhimurium* plays a critical role in the bacterium’s survival during oxidative stress (Liu et al., 2007), and USP in *Listeria monocytogenes* confers protecting activity for salt, acidity, and oxidative stress (Esvan et al., 2000; Gomes et al., 2011). Rv2623, a USP isoform in *Mycobacterium tuberculosis*, has an important role in mycobacterial growth and leads to chronic infection of humans (Drumm et al., 2009; Glass et al., 2017). Although physiological functions of bacterial USP are demonstrated in diverse cellular physiology and pathogenicity, the biochemical and molecular mechanism of most USPs remain largely unknown.

### USPs and Structural Diversity in Plants

Similar to bacterial USPs, diverse forms of USP have been identified from different plant sources by searching the internet database, Ensembl Plants^1^, and found 2,141 USPs (Table 1). All the proteins contain at least one USP domain and other catalytic motifs, which are differentially expressed in specific tissues, organs, and developmental stages or under different stress conditions (Li et al., 2010; Wang et al., 2017). The result suggests that plant USPs exert their distinctive function in specific tissues and developmental stages under particular stress conditions. Numbers of USPs found in various plant species are summarized in Table 1. The largest number of USPs are found in *Brassica napus* which contains 142 USPs. And the genomes of *Triticum aestivum, Brassica rapa*, *Solanum lycopersicum*, *Solanum tuberosum*, *Oryza sativa japonica*, *Vitis vinifera*, and *Zea mays* have 123, 71, 42, 41, 38, 33, and 43 *USP* genes, respectively. Although numerous numbers of *USP* genes and their wide distribution in diverse plant species implicate their importance in plant growth and development, the physiological and biochemical functions of plant USPs remain largely unknown. Especially, from the fact that many USPs are found in crop plants, it may be proposed that *USP* genes might be multiplied during the domestication procedures. The evolutionary pathway of plant USPs, as well as their duplication and functional specificity, should be further studied to determine why plants have so many USPs.

**TABLE 1:** Numbers of USPs found in different plant species^*^.

**Plant Sources**	**Number of USPs in plants**	**Plant Sources**	**Number of USPs in plants**
*Ostreococcus lucimarinus*	5	*Leersia perrieri*	41
*Chlamydomonas reinhardtii*	7	*Oryza meridionalis*	41
*Physcomitrella patens*	17	*Solanum tuberosum*	41
*Dioscorea rotundata*	20	*Arabidopsis lyrata*	42
*Lupinus angustifolius*	21	*Hordeum vulgare*	42
*Amborella trichopoda*	25	*Oryza glumipatula*	42
*Selaginella moellendorfii*	26	*Solanum lycopersicum*	42
*Triticum urartu*	27	*Sorghum bicolor*	42
*Oryza longistaminata*	30	*Manihot esculenta*	43
*Aegilops tauschii*	31	*Prunus persica*	43
*Corchorus capsularis*	32	*Zea mays*^†^	43
*Cucumis sativus*	32	*Arabidopsis thaliana*^†^	44
*Vitis vinifera*	33	*Oryza nivara*	44
*Beta vulgaris*	34	*Nicotiana attenuata*	46
*Brachypodium distachyon*	34	*Oryza punctata*	46
*Oryza brachyantha*	34	*Oryza rufipogon*	46
*Oryza glaberrima*	37	*Oryza sativa indica*	46
*Setaria italica*	37	*Gossypium raimondii*	53
*Theobroma cacao*	37	*Musa acuminata*	58
*Trifolium pratense*	37	*Populus trichocarpa*	62
*Oryza sativa japonica*^†^	38	*Glycine max*	70
*Phaseolus vulgaris*	39	*Brassica rapa*	71
*Medicago truncatula*	40	*Brassica oleracea*	74
*Oryza barthii*	40	*Triticum aestivum*	123
*Helianthus annuus*	41	*Brassica napus*	142

^*^The number of USPs in each plant species was obtained from the Ensembl Plants database (http://plants.ensembl.org/index.html). ^†^Phylogenetic tree of the three representative plant USPs (Oryza sativa japonica, Zea mays, Arabidopsis thaliana) is constructed and presented in Figure 3B (Oryza sativa japonica) and in Supplementary Figures 1, 2 (Zea mays, Arabidopsis thaliana).

**FIGURE 3:**
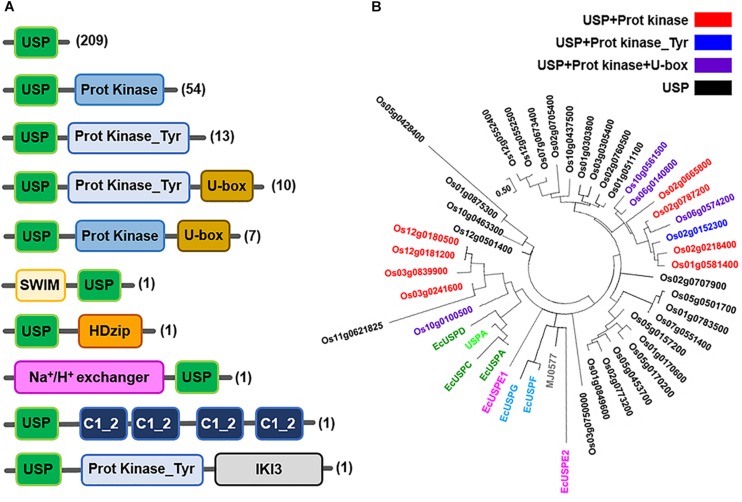
Molecular structures of diverse plant USPs and the phylogenetic tree of 38 USPs in *Oryza sativa japonica.* **(A)** Molecular structures of diverse USPs in plant sources (*Brachypodium distachyon*, *Setaria italic, Oryza sativa japonica, Medicago truncatula, Zea mays, Arabidopsis thaliana* and *Populus trichocarpa*) containing only a USP domain or USP domains fused with other catalytic motifs that are obtained from Ensembl Plants database (http://plants.ensembl.org/index.html). The domain architectures of different USPs are obtained from the Uniprot database (http://www.uniprot.org/), and domains are predicted using the InterPro database (http://www.ebi.ac.uk/interpro). Numbers of USPs having the specific type of molecular structure in plants are indicated at the left side of the figure in parenthesis. Each type of domain and motif is represented by different color-boxes. **(B)** Phylogenetic tree of the 38 USPs in *Oryza sativa* extracted from the Ensembl Plants database (http://plants.ensembl.org/index.html). The tree was constructed with USP domains of 38 *Oryza sativa* USPs after deleting all other domain sequences with the use of Maximum Likelihood method in MEGA7 (Kumar et al., 2016). *E. coil* USPs, *Methanocaldococcus jannaschii* MJ0577 and *Haemophilus influenza* USPA are included as references in the phylogenetic tree. The tree is drawn to scale, with branch lengths measured in the number of substitutions per site. The analysis involves 53 amino acid sequences. All positions containing gaps and missing data are eliminated. There are a total of 57 positions in the final dataset. Black, red, blue and violet color labeled USP proteins contain USP domain only, USP + Protein Kinase motifs USP + Protein Kinase_Tyr motif and USP + Protein Kinase + U-box motif, respectively. EcUSPA, EcUSPC and EcUSPD are represented in green; EcUSPF and EcUSPG by sky blue; EcUSPE1 and EcUSPE2 by magenta; MJ0577 by gray; USPA by light green colors.

Like the bacterial USPs, plant USPs have diverse functional motifs and a variety of structural characteristics. USPs in seven representative plant species including *Oryza sativa*, *Medicago truncatula*, *Zea mays*, *Brachypodium distachyon*, *Setaria italica*, *Populus trichocarpa*, and *Arabidopsis thaliana* are shown in Figure 3A. The most common type of USP in the plants has only a single USP domain, but the other proteins additionally contain a variety of other functional motifs. Using their amino acid sequences, phylogenetic tree of the three representative plant USPs in *Oryza sativa*, *Zea mays* and *Arabidopsis thaliana* is derived (Figure 3B and Supplementary Figures 1, 2). The phylogenetic tree of plant USPs strongly suggests that the functional diversity of plant USPs is much greater than those of bacterial USPs, because the latter is clustered within a very narrow range of evolutional tree. The other catalytic motifs found in plant USPs include serine/threonine kinase, tyrosine kinase, U-box, SWI2/snf2 and Mudr (SWIM)-zinc finger, HomeoDomain leucine zipper (HDzip), cation exchanger, C1 motif of Insensitive to Killer toxin3 (IKI3). The catalytic motifs of plant USPs may be derived over the course of evolution during the selective pressure against diverse stresses, which leads to the fusion of different catalytic motifs with a USP domain. The process provides plants for multiple strategies to protect them from foreign stresses (Kerk et al., 2003). The fusion of a cation exchange motif with the USP domain endows plants with protection from sodium toxicity. The cation exchange motif added to the USP domain pumps the sodium out from plant cells and prevents its accumulation. Fusion of protein kinase motif with a USP domain enables the protein to bind and utilize ATP and performs the energetically unfavorable reaction. The combination of such functional motifs with a USP domain at their amino terminal or carboxy terminal region explicitly suggests that divergent USPs have evolved to play specific roles under particular stress conditions, which protects plants from kaleidoscopic circumstances. The extensive shuffling of USP domain with a wide variety of functional motifs has provided plants with diverse sophisticated tactics for their survival under variable external conditions.

### Physiological Significance of Plant USPs in Biotic and Abiotic Defense Signaling

To protect plants from myriad of biotic and abiotic stresses caused by environmental stimuli, they have to develop highly advanced and sophisticated systems and devices (Conrath, 2011; Petrov et al., 2015; Martinez-Medina et al., 2016). Given the large numbers of plant USPs, it is reasonable to suppose that the proteins play crucial roles in diverse aspects of plant physiology and metabolism. Their functional diversity originated from the variety of other catalytic motifs fused with the USP domain is critically important for plant stress resistance. Although, only a little information is available on the biochemical and functional properties of plant USPs yet, several cases are introduced on the physiological importance of individual USPs related to plant stress responses (Table 2).

**TABLE 2:** Physiological functions of USPs identified from different plant species.

**Plant species**	**Name of USPs (accession number of the genes)**	**Physiological functions**	**References**
*Arabidopsis thaliana*	AtUSP (At3g53990)	Molecular chaperone under heat and oxidative stress	Jung et al., 2015
		RNA chaperone under cold stress	Melencion et al., 2017
	HRU1 (At3g03270)	Modulates ROS production under anoxia	Gonzali et al., 2015
*Solanum pennellii*	SpUSP (SGN-U214690)	ABA-induced stomatal movement, increase in photosynthetic efficiency, and alleviation of oxidative stress	Loukehaich et al., 2012
*Solanum lycoperiscus*	SlRd2 (SGN-U567775)	LiCl tolerance in yeast, Suppression of SlCipk6-mediated oxidative stress in plants	Gutierrez-Beltran et al., 2017
*Salicornia brachiata*	SbUSP (KF164282)	Enhancing the plant growth, alleviation of ROS build-up, and maintenance of ion homeostasis	Udawat et al., 2016
*Oryza sativa*	OsUsp1 (Os07g0673400)	Ethylene-mediated stress adaptation in rice	Sauter et al., 2002
*Salvia miltiorrhiza*	SmUSP1 (MF614040)	Enhancing the tolerance against salt, and heat stress in *E. coli*	Wang et al., 2017
	SmUSP8 (MF614047)		
	SmUSP27 (MF614066)		
*Astragalus sinicus*	AsD243 (DQ199645)	Functioning in the nodulation process in plant roots	Chou et al., 2007

**FIGURE 4:**
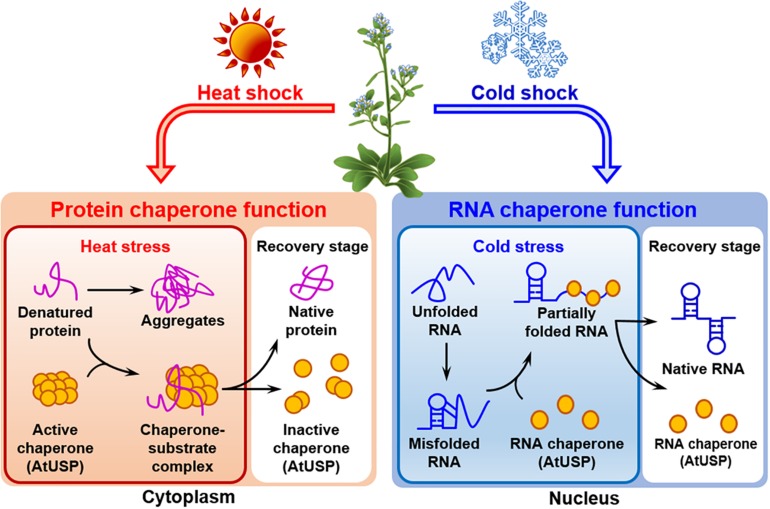
Identified functions of Arabidopsis USPs in response to temperature stresses. Arabidopsis USP, AtUSP, functions as a protein chaperone and an RNA chaperone under high- and low-temperature conditions, respectively. (Left panel) The protein chaperone function of AtUSP protects crucial intracellular substrates from heat shock-mediated aggregation working at the plant cytoplasm. During the process, protein structure of AtUSP changes from a small molecular species to oligomeric complexes in response to heat shock. The structural change enables the protein to get the protein chaperone function. (Right panel) AtUSP acts as an RNA chaperone under cold stress condition working at the plant nucleus. AtUSP can restore the cold-mediated unfolded or over-stabilized non-functional RNAs to their native forms of functional RNAs to serve them for protein translation.

All the forty-four USPs found in Arabidopsis genome contain an ATP-binding site and exhibit a high sequence homology to that of 1MJH protein family (Kerk et al., 2003). The proteins have diverse functions in protecting plants from different stresses as follows; HRU1, an Arabidopsis USP, regulates the intracellular level of hydrogen peroxide (H_2_O_2_) under hypoxic condition and transduces the oxygen-deficient signal into the downstream defense signaling pathway (Gonzali et al., 2015). Thus, at low oxygen concentration, it induces dissociation of the cytosolic form of dimeric HRU1 into monomeric HRU1, which translocates into the plasma membrane to interact with its partner, ROP2-RbohD, and make the HRU1-ROP2-RbohD complex (Table 3). The complex increase the intracellular concentration of H_2_O_2_ and finely tunes H_2_O_2_ level, that enables the plants to recover from anoxia. Another type of Arabidopsis USP, AtUSP, was identified from the cytosolic extracts treated with heat shock or oxidative stress. Under optimum conditions, AtUSP exists as diverse forms including monomer, dimer, trimer, and oligomeric complexes. When plants are exposed to heat shock and/or oxidative stress, the intracellular redox status is changed to make a shift of AtUSP from low molecular weight species to a high molecular weight complex (Jung et al., 2015). The protein structure of AtUSP existed as an inactive monomer or dimer under optimum conditions can be switched into a high oligomer complex in response to external stress, which provides the chance of the protein to acquire a novel function of ‘molecular chaperone’ working at the plant cytoplasm (Figure 4, left panel). Then, the holdase chaperone function of AtUSP allows it to prevent denaturation of intracellular core macromolecules from heat shock or oxidative stress. This action resembles the general properties of most heat shock proteins. The heat shock-mediated functional transition of AtUSP to a holdase chaperone ensures transgenic plants over-expressing AtUSP to have a strong resistance to heat or oxidative stress.

**TABLE 3:** Interaction partners of USPs identified from plant sources and their functions.

**Plant species**	**USP**	**Target proteins**	**Function of target protein**	**References**
*Arabidopsis thaliana*	HRU1 (At3g03270)	GTPase ROP2 (At1g20090)	• ROS generation • Inhibition of ABA- and CO_2_- induced stomatal closure	Gonzali et al.,2015
				Hwang et al.,2011
		RbohD (At5g47910)	• ROS generation in plant defense response	Gonzali et al.,2015
				Torres et al.,2002
		Thioredoxin h1 (At3g51030)	• Functioning as disulfide reductase, protein chaperone • Regulation of AtCDK21 (Calcium-dependent protein kinase21) activity	Gonzali et al.,2015
				Ueoka-Nakanishi et al.,2013
				Jung et al.,2013
	AtUSP (At3g53990)	Thioredoxin h1 (At3g51030)	• Functioning as disulfide reductase, protein chaperone • Regulation of AtCDK21 activity	Gonzali et al.,2015
				Ueoka-Nakanishi et al.,2013
				Jung et al.,2013
	At3g17020	Thioredoxin h1 (At3g51030)	• Functioning as disulfide reductase, protein chaperone • Regulation of AtCDK21 activity	Gonzali et al.,2015
				Ueoka-Nakanishi et al.,2013
				Jung et al.,2013
*Solanum pennellii*	SpUSP (SGN-U214690)	AnnSp2 (Sopen04g025030.1)	• Drought and salt tolerance by modulation of ABA synthesis and elimination of ROS	Loukehaich et al.,2012
				Ijaz et al.,2017
*Solanum lycoperiscum*	SlRd2 (SGN-U567775)	SlCipk6 (SGN-U271168)	• ROS generation during effector-triggered immunity	Gutierrez-Beltran et al.,2017
				De la Torre et al.,2013

In addition to heat shock-mediated post-translational modification of AtUSP acting as a protein chaperone, mRNA level of *AtUSP* was significantly enhanced at low temperature, suggesting that AtUSP might have another specific role in cold stress (Melencion et al., 2017). From the subcellular localization of AtUSP in cold condition and investigation of RNAs stability with or without the presence of AtUSP, it can be concluded that the protein also gets another function of ‘RNA chaperone’ under cold condition functioning at the plant nucleus (Figure 4, right panel) (Kang et al., 2013; Melencion et al., 2017). In fact, low temperature induces over-stabilization or misfolding of RNA molecules, which are inactive to serve as a template RNAs (Lorsch, 2002; Kang et al., 2013). Then the RNA chaperone function of AtUSP enables the unwinding of over-stabilized RNA molecules and refolding them into their active structures, to provide them as RNA templates for their translation. Consequently, overexpression of AtUSP protects the transgenic plants from cold and freezing conditions. The physiological roles of AtUSP are therefore critical for protecting plants from both high and low temperatures, playing dual functions in plants to adapt for the environmental temperature fluctuation (Jung et al., 2015; Melencion et al., 2017). The result is also demonstrated in chilling tolerance of grapefruit (*Citrus paradisi* cv. Star ruby), that is achieved by altering the expression of its *USP*. The gene can be induced by exposing the fruit to a short pre-treatment with hot water and then briefly rinsing and brushing, which reduces injuries during subsequent chilling (Sapitnitskaya et al., 2006).

Apart from the temperature-associated function of AtUSP, another USP in *Salicornia brachiata* (SbUSP) is shown to involve in abiotic stress resistance (Udawat et al., 2016). Ectopic expression of *SbUSP* in tobacco plants significantly enhances salt tolerance, exhibiting an increased osmotic stress resistance through the removal of intracellular ROS. Under the salt and osmotic stress conditions, the osmosensor recognizes the cellular Na^+^ level and activates the specific protein kinase involved in salt signaling. This protein kinase then phosphorylates serine and threonine residues of SbUSP. Next, the phosphorylated SbUSP activates the expression of downstream target genes, which causes an accumulation of osmoprotectant, alleviates intracellular ROS build-up, and protects plants from salt stress. SpUSP from tomato (*Solanum pennellii*) and GhUSP from cotton (*Gossypium hirsutum* L.) are also induced by salt stress, suggesting that the USPs play crucial roles in plant salt-stress tolerance (Loukehaich et al., 2012; Li et al., 2015). Besides the salt stress, *SpUSP* gene is noticeably induced by the treatment of drought, heat/cold shock, treatment of paraquat, wounding, and phytohormone (abscisic acid, gibberellic acid, and ethylene) (Loukehaich et al., 2012). In addition, transcript level of *SlRd2* mRNA, another *USP* gene in tomato (*Solanum lycopersicum*), is critically enhanced by the treatment of salt and LiCl, suggesting that the physiological function of SIRd2 might be involved in salt and osmotic stress tolerance in plants (Gutierrez-Beltran et al., 2017). Thus, overexpression of SIRd2 in *S. cerevisiae* (BY4741) strongly exhibits osmotic tolerance as well as the expression of *SlRD2* gene in *uspA E. coli* mutant (TN3151), displaying a highly sensitive phenotype to oxidative stress and clearly complements the mutant properties, which restores bacterial viability in the presence of 5 mM H_2_O_2_. Since the SIRd2 function is probably associated with the removal of intracellular ROS in plants, the expression of SlCipk6 and GFP-SIRd2 in tobacco plants decreases the ROS level. To carry out the assignment, SlCipk6 (Calcineurin B-like interacting protein kinase) phosphorylates the dimeric form of SIRd2, which negatively regulates the SlCipk6-mediated ROS production (Table 3) (Gutierrez-Beltran et al., 2017). In transgenic tomato overexpressing *SpUSP*, abscisic acid level is elevated under drought condition and induces stomatal closure to reduce water loss, that endows plants with drought tolerance and improved photosynthetic efficiency. During the procedure, SpUSP is shown to interact with annexin (AnnSp2) known as a target of calcium signaling in eukaryotes analyzed by yeast two-hybrid and bimolecular fluorescence complementation (BiFC) techniques (Table 3) (Loukehaich et al., 2012). Annexin plays prominent roles in abiotic and biotic stress resistance in plants (Jami et al., 2008; Konopka-Postupolska et al., 2009; Szalonek et al., 2015; Konopka-Postupolska and Clark, 2017). Therefore, similar to the plants overexpressing SpUSP, the overexpression of AnnSp2 in tomato critically enhances their drought tolerance through the stomatal closure, build-up of ABA and chlorophyll contents, reduction in water loss, elimination of ROS, decreasing the level of lipid peroxidation, increase in proline concentration and antioxidant activities (Ijaz et al., 2017).

Furthermore, transcription of *GaUSP1* and *GaUSP2* in *Gossypium arboretum* is induced by drought stress, indicating that these two GaUSPs function in the control of intracellular water content (Maqbool et al., 2008, 2009). Treatment with various stress-inducing factors, such as salt, dehydration, darkness, heavy metals, and phytohormones covering abscisic acid and gibberellic acid strongly increases the activity of cotton *USP* promoters. Activity of a 949 bp fragment of the cotton *USP* promoter is significantly increased in transgenic tobaccos during the stress treatment, as shown in *USP* mRNA levels (Zahur et al., 2009). Drought stress also upregulates the expression of *USP* genes in Amor cork tree (*Phellodendron amurense*) and pigeon pea (*Cajanus cajan* L.) (Wang et al., 2008; Sinha et al., 2015). Furthermore, in rice (*Oryza sativa*), OsUSP1 containing the conserved ATP-binding residues regulates phytohormone signaling to increase stress resistance and protects plants during submergence in water by regulating ethylene concentration in cells (Sauter et al., 2002).

Besides their roles in abiotic stress resistance, plant USPs also participate in defense response against pathogenic attack. Infection of the Chinese Milk Vetch (*Astragalus sinicus*) roots with the nodule-inducing leguminous bacterium, *Mesorhizobium huakuii*, results in an increased expression of *USP* gene *AsD243*, which suggests that AsD243 plays a role in nodule development (Chou et al., 2007). Following elicitation of Arabidopsis cells by treating the *Phytophthora infestans* zoospores or bacterial eliciting peptide, flagellin-22, two USPs including AtPHOS32 and AtPHOS34 are phosphorylated by mitogen-activated protein kinases (MAPKs). AtPHOS32, a substrate of MAP kinases 3 and 6, involves in pathogen defense signaling. Phosphorylated USPs thus appear to activate defense signaling in plants to provide protection against pathogenic attacks (Lenman et al., 2008; Merkouropoulos et al., 2008). All these results strongly suggest that plant *USP* genes are upregulated in response to diverse external stresses. As the wider biochemical functions and molecular properties of USPs remain largely obscure, future studies should focus on the identification of their roles in protecting plants against particular biotic/abiotic stress and also investigate their molecular mechanisms in more detail with the use of double or triple *usp* mutants, or mutants lacking specific catalytic motifs.

### Redox Regulation of Plant USPs

Plants protect themselves from diverse internal and external stresses, including heat shock, freezing stress, salt, heavy metal toxicity, flooding, drought, and biotic pathogens, using complicated and dynamic strategies that are principally regulated by redox signaling (Chi et al., 2013; Geigenberger et al., 2017). Plants have developed delicate redox signaling systems that sense internal redox changes and respond by activating specific intracellular redox-mediated defense signaling pathways (Gonzalez-Bosch, 2018; Noctor et al., 2018). ROS, the by-products of physiological O_2_ metabolism including H_2_O_2_, superoxide anions (O_2_^⋅−^), hydroxyl radical (OH⋅), and singlet oxygen (O_2_), are precisely controlled by enzymatic and non-enzymatic antioxidant defense systems (Nita and Grzybowski, 2016). They induce oxidative damage of cells and eventually result in cell death (Gill and Tuteja, 2010; You and Chan, 2015). A well-characterized typical redox system in plants includes glutaredoxin/thioredoxin proteins, which play a central role in the regulation of carbon metabolism and photosynthesis (Gutsche et al., 2015; Geigenberger et al., 2017; Nikkanen et al., 2017). In plants, redox proteins consist of multigene families with a large number of potential target molecules related to diverse aspects of cellular metabolism (Delorme-Hinoux et al., 2016; Geigenberger et al., 2017; Mata-Perez and Spoel, 2019). The proteins are core components of plant defense signaling pathways and act as a dynamic linker between stress perception and physiological responses. Redox proteins modulate target enzyme’s activity by post-translational modification through the oxidation and reduction of their catalytic Cys residues in response to ROS changes. The 2-Cys peroxiredoxins (2-Cys Prxs) are ROS sensors that participate in redox signaling through their structural switching between the monomer form and oligomeric complexes (Konig et al., 2002; Jang et al., 2004; Perkins et al., 2015). Other redox proteins including thioredoxin-3, AtTDX, and AtNTRC, exhibit multiple functions in response to environmental stimuli *via* redox-dependent processes (Lee et al., 2009; Mayer, 2012; Chae et al., 2013; Chi et al., 2013). Besides the redox proteins, there are different transcriptional factors, such as Rap2.4a and AtbZIP16 and Non-expresser of Pathogenesis Related gene 1 (NPR1), regulating the expression of stress-responsive genes or antioxidant enzymes by redox- and structure-dependent manner (Shaikhali et al., 2008, 2012; Montrichard et al., 2009; Chi et al., 2013).

In particular, the chaperone function and structural change of AtUSP is altered by treatment with dithiothreitol (DTT) and/or H_2_O_2_ (Jung et al., 2015). AtUSP with multimeric complexes in optimum conditions changed into a monomer, accompanying with a decrease in its chaperone activity by DTT treatment. In reverse, the monomer form of AtUSP shifts into high oligomeric complex by H_2_O_2_, together with an increase in its chaperone activity. These results suggest that functional and structural switching of AtUSP is regulated by redox-dependent manner like other redox proteins, transcription factors and co-activators. The functions of USPs identified from plant sources are involved in modulating ROS concentration produced by diverse environmental stress (Table 2). In Arabidopsis, the interaction of three USPs (HRU1, AtUSP, and At3g17020) with a redox partner, thioredoxin-h1, have been determined by affinity chromatography, yeast two-hybrid analysis, and BiFC assays (Table 3) (Ueoka-Nakanishi et al., 2013; Gonzali et al., 2015). The thioredoxin-h1 is known to regulate the activity of calcium-dependent protein kinase 21 (AtCPK21) and reactivates the oxidized AtCPK21 under oxidative stress with a redox regulation (Ueoka-Nakanishi et al., 2013). Since the HRU1 and AtUSP are target proteins of thioredoxin-h1 as well as their amino acid sequences contain two conserved cysteine residues, the results provide a strong possibility on the involvement of USPs in redox regulation. From the results, it can be clearly demonstrated that the structural switching of AtUSP is induced by redox change in response to external stress, accompanying with its functional alteration. The importance of the cysteine residues in this redox-dependent regulation of USPs requires further investigation.

### Biotechnological Application of USPs for the Development of Stress-Tolerant Valuable Crops

Controlling the expression of specific plant genes results in the activation of numerous biological signaling pathways and intracellular metabolic networks that influence plant growth, development, physiology, and productivity (Finkel, 2011). It is well known that, in plants, the complex signaling networks involved in stress responses mutually regulate each other’s activities through the cross-talk involved in their communication and translational and post-translational modification. Plant USPs participate in a number of cellular metabolism to regulate defense systems against diverse external stresses. Regulating the expression of *USP* genes may therefore provide a powerful strategy for the development of stress-tolerant varieties of crop plants. The success of such an approach requires detailed understanding of USP functions in molecular basis underlying the stress tolerance responses in plants. As plant USPs have diverse roles in defense responses in response to ever-changing environmental stresses, it may be necessary to manipulate their expression to produce highly valuable, stress-tolerant crops that have valuable application in the agricultural fields. Physiological importance of USPs in plants strongly support the idea that control of *USP* gene expression in important crop plants in combination with other techniques, such as molecular breeding and genetic engineering may produce novel and high productive crop varieties (Jung et al., 2015; Udawat et al., 2016; Gutierrez-Beltran et al., 2017; Melencion et al., 2017). Thus, under conditions involving unfavorable environmental stresses, such as climate change, extreme temperatures, and other severe environmental problems, projects applying an understanding of *USP* gene function are likely to be highly important to the preparation of future varieties of stress-tolerant crops.

## Conclusion

This review highlights the important roles played by USPs in the survival of all living organisms, including bacteria, Archaea, fungi, plants, and metazoans, in the face of diverse environmental stresses. Despite the great importance of USPs, their molecular properties remain largely unknown. They are widely distributed across different cell types and species, indicating their significance in plant tissues, organs, and physiology. In plants, their functions include acting as protein chaperone and RNA chaperone, nucleotide binding, and prevention of hypoxia, and thus USPs offer protection from a wide range of external stresses. As USPs have versatile structures, resulting from the fusion of the USP domain with many other catalytic motifs, it is highly likely that these proteins are involved in multiple reactions and diverse cellular processes under stressful conditions. Furthermore, the other catalytic motifs allow functional diversity by enabling structural switching from small molecular species to high molecular complexes in response to external stresses. Exposure to heat shock and oxidative shock, in particular, induces the formation of high molecular complexes that function as protein chaperones, preventing denaturation of crucial intracellular molecules due to thermal stress. As there are many USPs in plants, it will be necessary to unravel the functional specificity of individual USPs in different species. The greatest challenge facing investigators of plant USPs is the determination of their physiological and biochemical functions in relation to plant metabolism. Uncovering these functions may unlock new biotechnological applications and lead to the development of valuable, stress-resistant crops. Through an applied understanding of the function of USPs, it may be possible to develop novel varieties with high productivity under unfavorable growth conditions. This should provide a focus for future investigation.

## Author Contributions

YC, MK, W-YK, D-JY, and SL made a substantial contribution to the conception, design, and writing of this manuscript. SK, HO, EL, JP, KP, SW, SB, SP, HC, and CK collected USP gene and protein datasets from plant sources and designed the figures and tables.

## Conflict of Interest Statement

The authors declare that the research was conducted in the absence of any commercial or financial relationships that could be construed as a potential conflict of interest.

## Footnotes

1 http://plants.ensembl.org/
