# Supplementary material for: The Physiological Functions of Universal Stress Proteins and Their Molecular Mechanism to Protect Plants From Environmental Stresses
Source: Front Plant Sci. 2019 Jun 5;10:750. doi: 10.3389/fpls.2019.00750 (PMC6560075; doi:10.3389/fpls.2019.00750)

Supplementary Figure 1. Phylogenetic tree of 43 USPs in *Zea mays*

Phylogenetic tree of the *43* USPs in *Zea mays* extracted from the Ensembl Plants database (http://plants.ensembl.org/index.html). The tree was constructed with USP domains of 43 USPs of *Zea mays* after deleting all other domain protein sequences with the use of Maximum Likelihood method in MEGA7 (Kumar et al., 2016). *E. coil* USPs, *Methanocaldococcus jannaschii* MJ0577 and *Haemophilus influenza* USPA are included as references in the phylogenetic tree. The tree is drawn to scale, with branch lengths measured in the number of substitutions per site. The analysis involves 53 amino acid sequences. All positions containing gaps and missing data are eliminated. There are a total of 57 positions in the final dataset. Black, red, blue and violet color labelled USP proteins contain USP domain only, USP + Protein Kinase motifs USP + Protein Kinase_Tyr motif and USP + Protein Kinase + U-box motif, respectively. EcUSPA, EcUSPC and EcUSPD are represented in green; EcUSPF and EcUSPG by sky blue; EcUSPE1 and EcUSPE2 by magenta; MJ0577 by gray; USPA by light green colors.

Supplementary Figure 2. Phylogenetic tree of 43 USPs in Arabidopsis thaliana

Phylogenetic tree of the *44* USPs in *Arabidopsis thaliana* extracted from the Ensembl Plants database (http://plants.ensembl.org/index.html). The tree was constructed with USP domains of 43 USPs of *Arabidopsis thaliana* after deleting all other domain protein sequences with the use of Maximum Likelihood method in MEGA7 (Kumar et al., 2016). *E. coil* USPs, *Methanocaldococcus jannaschii* MJ0577 and *Haemophilus influenza* USPA are included as references in the phylogenetic tree. The tree is drawn to scale, with branch lengths measured in the number of substitutions per site. The analysis involves 53 amino acid sequences. All positions containing gaps and missing data are eliminated. There are a total of 57 positions in the final dataset. Black, red, blue and violet color labelled USP proteins contain USP domain only, USP + Protein Kinase motifs USP + Protein Kinase_Tyr motif and USP + Protein Kinase + U-box motif, respectively. EcUSPA, EcUSPC and EcUSPD are represented in green; EcUSPF and EcUSPG by sky blue; EcUSPE1 and EcUSPE2 by magenta; MJ0577 by gray; USPA by light green colors.

Reference

1. Kumar, S., Stecher, G., and Tamura, K. (2016). MEGA7: Molecular Evolutionary Genetics Analysis Version 7.0 for Bigger Datasets. Mol. Biol. Evol. 33**,** 1870-1874.

Supplementary Figure 1

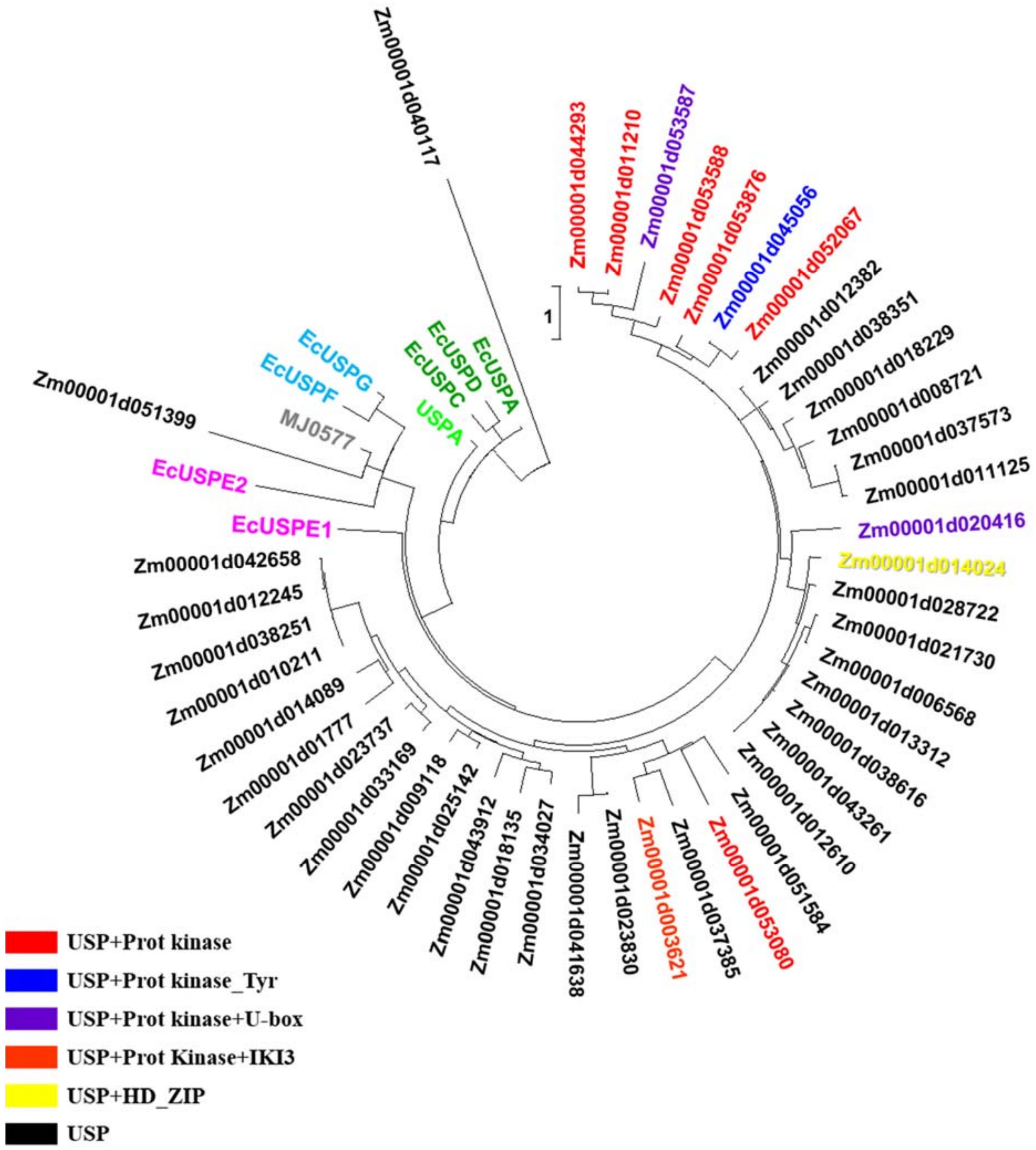

Supplementary Figure 2

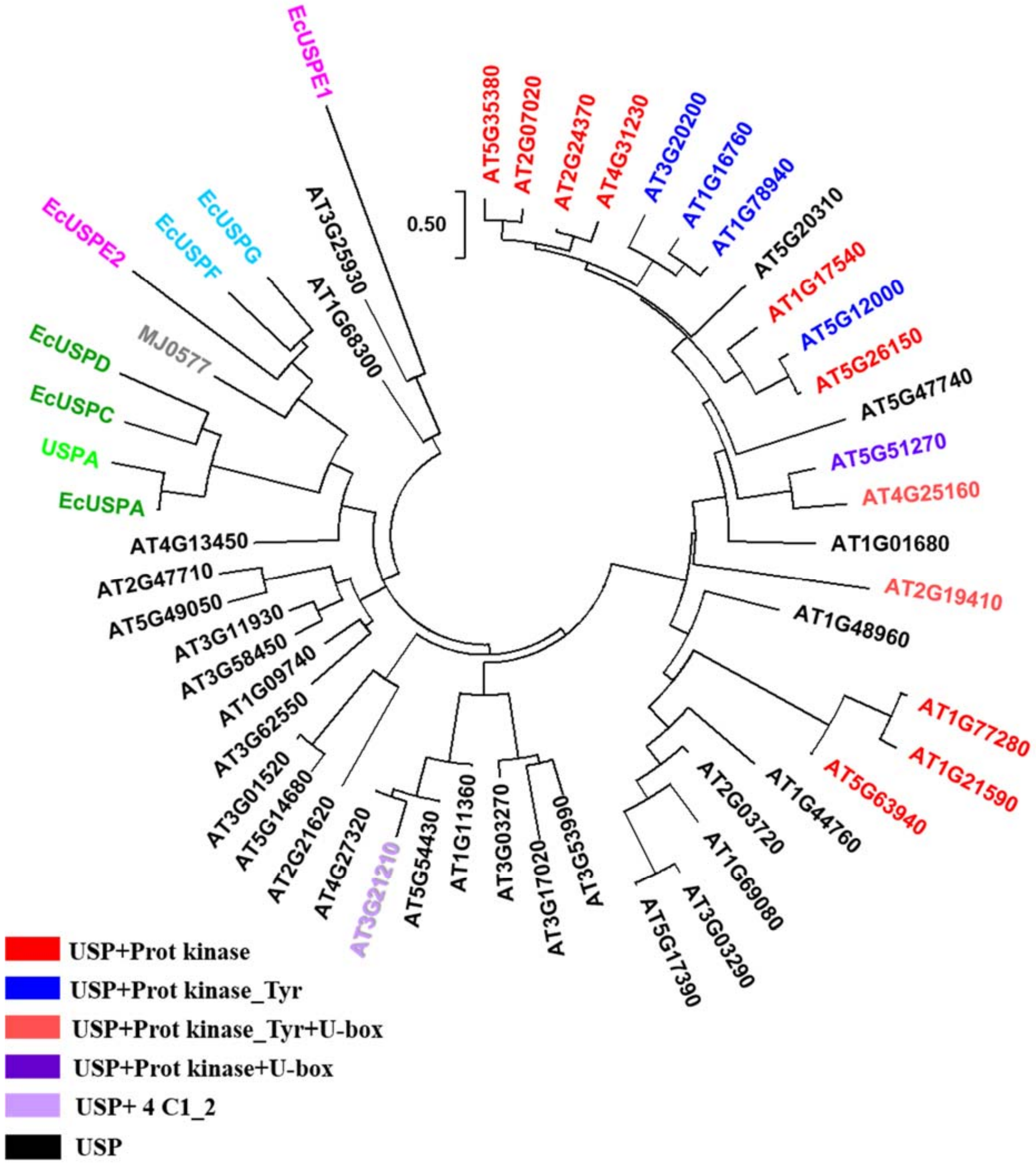

Supplement: Supplementary file 1 [file Data_Sheet_1.pdf]
